# Supplementary material for: Lactoferrin levels in cerebrospinal fluid exhibits isoform-specific associations with Alzheimer’s disease
Source: medRxiv. 2025 Oct 13:2025.09.24.25336349. Preprint. [Version 2] doi: 10.1101/2025.09.24.25336349 (PMC12622143; doi:10.1101/2025.09.24.25336349)
Supplement: Supplement 2 [file NIHPP2025.09.24.25336349v2-supplement-2.pdf]

medRxiv preprint doi: https://doi.org/10.1101/2025.09.24.25336349; this version posted October 13, 2025. The copyright holder for this preprint (which was not certified by peer review) is the author/funder, who has granted medRxiv a license to display the preprint in perpetuity.
It is made available under a CC-BY 4.0 International license .

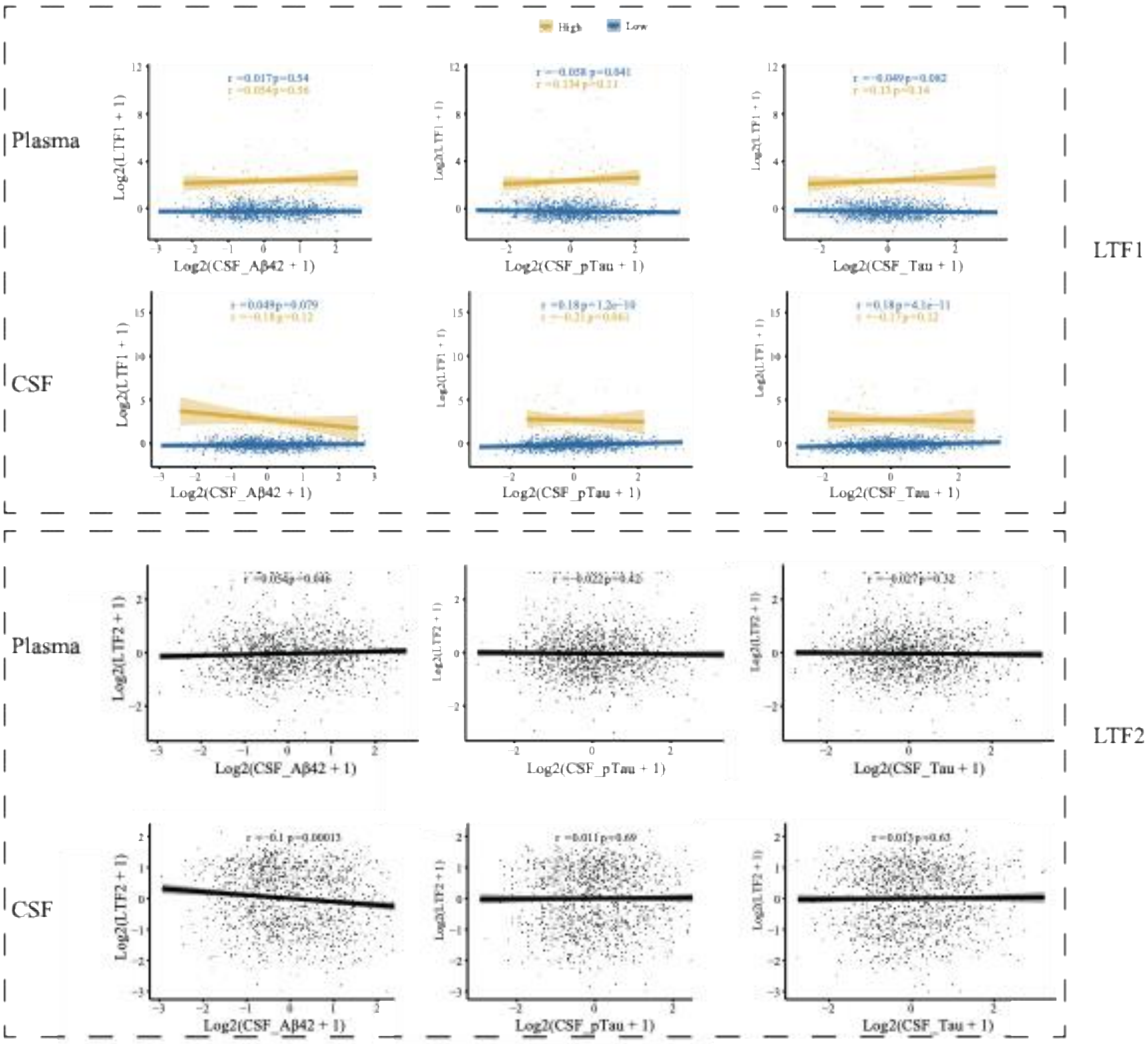

Fig. S1. Scatter plots showing the correlations between LTF1/LTF2 and AD biomarkers (Aβ42, p-tau, and t-tau).

The top row shows correlations with LTF1, and the bottom row with LTF2. LTF1 is stratified by LTF1 expression—LTF1-high (yellow) vs LTF1-low (blue). For LTF2, correlations were computed across all samples. Spearman correlation coefficients (r) and corresponding *p* values (*p*) are indicated in each panel.

medRxiv preprint doi: https://doi.org/10.1101/2025.09.24.25336349; this version posted October 13, 2025. The copyright holder for this preprint
(which was not certified by peer review) is the author/funder, who has granted medRxiv a license to display the preprint in perpetuity.
It is made available under a CC-BY 4.0 International license .

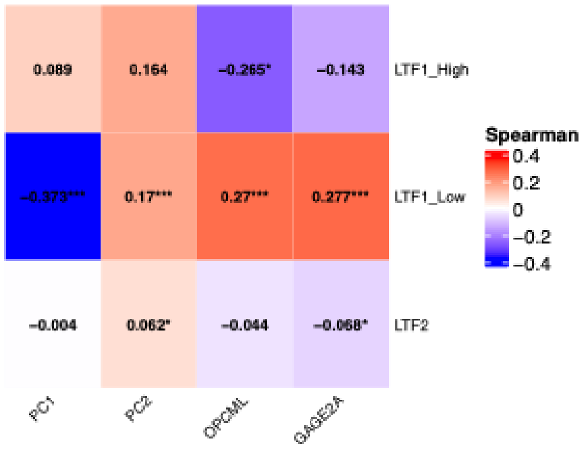

Fig. S2. **Spearman correlation heatmap between CSF LTF1-/LTF2 and the first two proteomic principal components (PC1–PC2) and reference genes (OPCML, GAGE2A).** Cells display Spearman's ρ; color encodes effect size. Significance: *, $p < 0.05$; **, $p < 0.01$; **, $p < 0.001$.

medRxiv preprint doi: https://doi.org/10.1101/2025.09.24.25336349; this version posted October 13, 2025. The copyright holder for this preprint (which was not certified by peer review) is the author/funder, who has granted medRxiv a license to display the preprint in perpetuity.
It is made available under a CC-BY 4.0 International license .

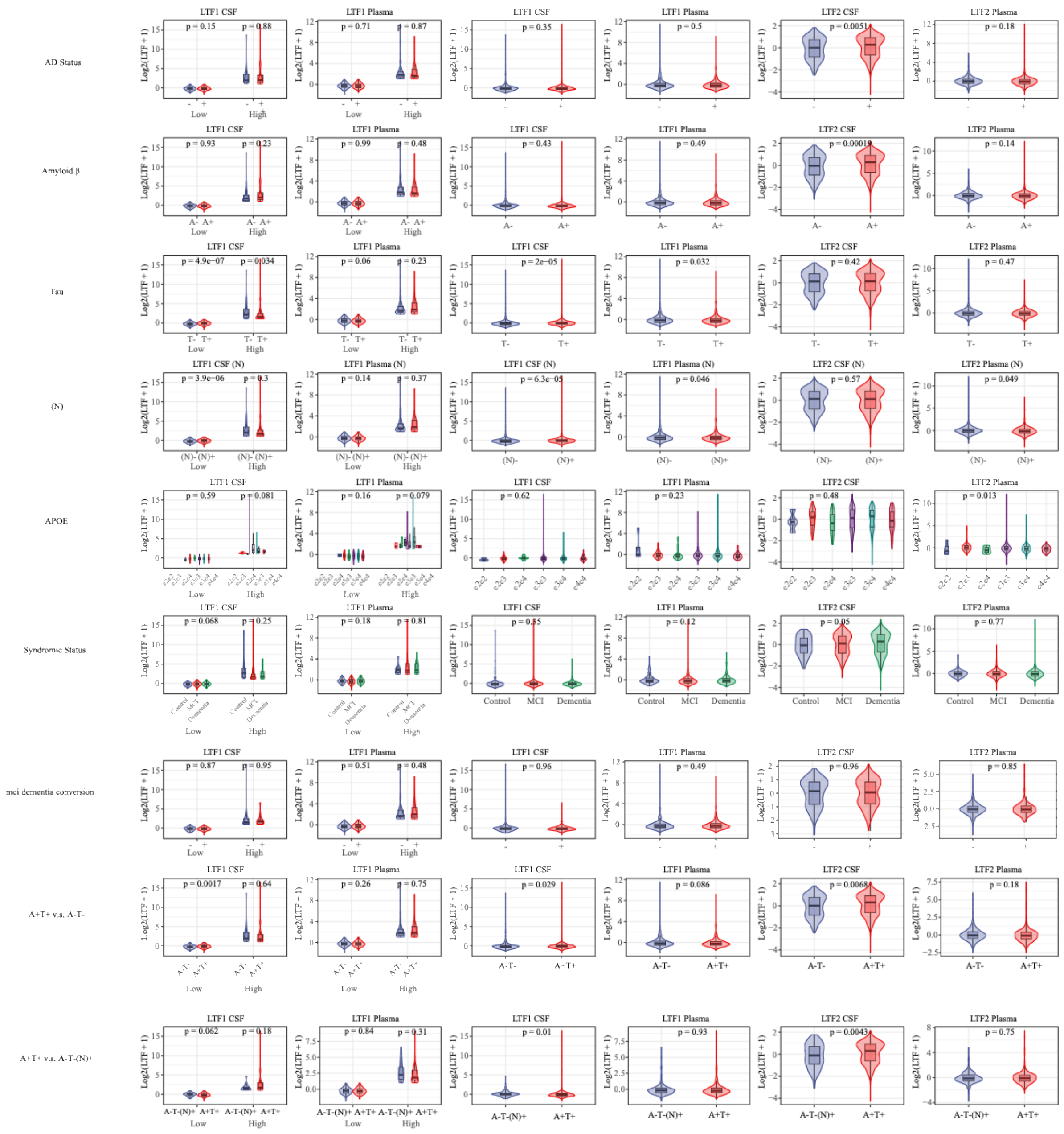

medRxiv preprint doi: https://doi.org/10.1101/2025.09.24.25336349; this version posted October 13, 2025. The copyright holder for this preprint
(which was not certified by peer review) is the author/funder, who has granted medRxiv a license to display the preprint in perpetuity.
It is made available under a CC-BY 4.0 International license .

Fig. S3. Comparison of LTF1 and LTF2 levels across AD biomarker profiles, clinical diagnosis, APOE genotypes, and longitudinal conversion status.

Each row represents a comparison between groups stratified by APOE genotype, dementia diagnosis, A/T/N biomarker positivity, or longitudinal conversion status. Columns 1 – 2 show CSF and plasma LTF1 levels stratified by LTF1 abundance (high, low) respectively; columns 3 – 4 show the CSF and plasma LTF1 levels before stratification. Columns 5 – 6 show CSF and plasma LTF2 levels. In each plot, different color were used to discriminate the status of diagnosis, AD biomarkers, APOE genotypes or conversion status respectively. Statistical comparisons were performed using the Kruskal-Wallis test.
